# Impact of body mass index in patients receiving atezolizumab plus bevacizumab for hepatocellular carcinoma

**DOI:** 10.1007/s12072-023-10491-3

**Published:** 2023-04-01

**Authors:** Mathew Vithayathil, Antonio D’Alessio, Claudia Angela Maria Fulgenzi, Naoshi Nishida, Martin Schönlein, Johann von Felden, Kornelius Schulze, Henning Wege, Anwaar Saeed, Brooke Wietharn, Hannah Hildebrand, Linda Wu, Celina Ang, Thomas U. Marron, Arndt Weinmann, Peter R. Galle, Dominik Bettinger, Bertram Bengsch, Arndt Vogel, Lorenz Balcar, Bernhard Scheiner, Pei-Chang Lee, Yi-Hsiang Huang, Suneetha Amara, Mahvish Muzaffar, Abdul Rafeh Naqash, Antonella Cammarota, Valentina Zanuso, Tiziana Pressiani, Matthias Pinter, Alessio Cortellini, Masatoshi Kudo, Lorenza Rimassa, David J. Pinato, Rohini Sharma

**Affiliations:** 1 Department of Surgery & Cancer, Imperial College London, Hammersmith Campus, Du Cane Road, London, W12 0HS UK; 2 Department of Biomedical Sciences, Humanitas University, Pieve Emanuele, Milan, Italy; 3 Division of Medical Oncology, Policlinico Universitario Campus Bio-Medico, Rome, Italy; 4 Department of Gastroenterology and Hepatology, Faculty of Medicine, Kindai University, Osaka, Japan; 5 Department of Oncology, Hematology and Bone Marrow Transplantation With Section of Pneumology, University Medical Center Hamburg-Eppendorf, Hamburg, Germany; 6 Department of Gastroenterology and Hepatology, University Medical Center Hamburg-Eppendorf, Hamburg, Germany; 7 Division of Medical Oncology, Department of Medicine, Kansas University Cancer Center, Kansas City, KS USA; 8 Division of Hematology/Oncology, Department of Medicine, Tisch Cancer Institute, Mount Sinai Hospital, New York, NY USA; 9 I. Medical Department, University Medical Center Mainz, Mainz, Germany; 10 Department of Medicine II (Gastroenterology, Hepatology, Endocrinology and Infectious Diseases), Faculty of Medicine, Freiburg University Medical Center, University of Freiburg, Freiburg, Germany; 11 University of Freiburg, Signalling Research Centers BIOSS and CIBSS, Freiburg, Germany; 12 German Cancer Consortium (DKTK), Partner Site, Freiburg, Germany; 13 Hannover Medical School, Hannover, Germany; 14 Division of Gastroenterology & Hepatology, Department of Internal Medicine III, Medical University of Vienna, Vienna, Austria; 15 Division of Gastroenterology and Hepatology, Department of Medicine, Taipei Veterans General Hospital, Taipei, Taiwan; 16 Institute of Clinical Medicine, School of Medicine, National Yang Ming Chiao Tung University, Taipei, Taiwan; 17 Division of Hematology/Oncology, East Carolina University, Greenville, NC USA; 18 Medical Oncology/TSET Phase 1 Program, Stephenson Cancer Center, University of Oklahoma, Norman, OK USA; 19 Medical Oncology and Hematology Unit, Humanitas Cancer Center, IRCCS Humanitas Research Hospital, Rozzano, Milan Italy; 20 Division of Oncology, Department of Translational Medicine, University of Piemonte Orientale, Novara, Italy

**Keywords:** Immunotherapy, Anti-programmed death-ligand, Anti-vascular endothelial growth factor, Checkpoint inhibitor, Obesity, Cirrhosis, Overall survival, Progression-free survival, Overweight, Non-alcoholic fatty liver disease

## Abstract

**Background:**

Atezolizumab plus bevacizumab (Atezo/Bev) is first line-treatment for unresectable hepatocellular carcinoma (HCC). Body mass index (BMI) has demonstrated predictive value for response to immunotherapy in non-HCC cancer types. Our study investigated the effect of BMI on safety and efficacy of real-life use of Atezo/Bev for unresectable HCC.

**Methods:**

191 consecutive patients from seven centres receiving Atezo/Bev were included in the retrospective study. Overall survival (OS), progression-free survival (PFS), overall response rate (ORR) and disease control rate (DCR) defined by RECIST v1.1 were measured in overweight (BMI ≥ 25) and non-overweight (BMI < 25) patients. Treatment-related adverse events (trAEs) were evaluated.

**Results:**

Patients in the overweight cohort (*n* = 94) had higher rates of non-alcoholic fatty liver disease (NAFLD) and lower rates of Hepatitis B compared to non-overweight cohort (*n* = 97). Baseline Child–Pugh class and Barcelona Clinic Liver Cancer stage were similar between cohorts, with lower rates of extrahepatic spread in the overweight group. Overweight patients had similar OS compared to non-overweight (median OS 15.1 vs. 14.9 months; *p* = 0.99). BMI did not influence median PFS (7.1 vs. 6.1 months; *p* = 0.42), ORR (27.2% vs. 22.0%; *p* = 0.44) and DCR (74.1% vs. 71.9%; *p* = 0.46). There were higher rates of atezolizumab-related fatigue (22.3% vs. 10.3%; *p* = 0.02) and bevacizumab-related thrombosis (8.5% vs. 2.1%; *p* = 0.045) in the overweight patients, but overall trAEs and treatment discontinuation were comparable between cohorts.

**Conclusion:**

Atezo/Bev has comparable efficacy in overweight HCC patients, with an increase in treatment-related fatigue and thrombosis. Combination therapy is safe and efficacious to use in overweight patients, including those with underlying NAFLD.

**Supplementary Information:**

The online version contains supplementary material available at 10.1007/s12072-023-10491-3.

## Background

Hepatocellular carcinoma (HCC) is the sixth most common cancer worldwide, and the fourth leading cause of cancer-related mortality [1]. The mainstay of advanced, unresectable HCC has been systemic therapy; previously sorafenib [2] and most recently lenvatinib [3]. However, the advent of immunotherapy has transformed the treatment landscape of advanced unresectable HCC [4]. Atezolizumab, an anti-programmed death-ligand (PD-L1) monoclonal antibody, and bevacizumab, an anti-vascular endothelial growth factor (VEGF) monoclonal antibody, have been used in combination for unresectable HCC. The IMbrave150 study demonstrated atezolizumab and bevacizumab (Atezo/Bev) combination therapy superiority over sorafenib for overall survival (OS) and progression-free survival (PFS) [5, 6]. Combination therapy extended median OS to 19.2 vs 13.4 months (hazard ratio (HR) 0.66, 95% confidence interval (CI) 0.52–0.85) and median PFS to 6.9 vs 4.3 months (HR 0.65, 95% CI 0.53–0.81) compared with sorafenib. In view of these findings Atezo/Bev now represents a first-line treatment option, along with single tremelimumab regular interval durvalumab (STRIDE) [7], for unresectable HCC [8].

Elevated body mass index (BMI) is an established risk factor for development HCC [9, 10]. However, the role of BMI in predicting HCC survival is less clear. Retrospective studies have varied in demonstrating increased BMI (overweight or obese defined as BMI ≥ 25) is associated with reduced [11–14], increased [15] or no change [16, 17] in OS in HCC patients undergoing treatments including surgical resection, trans-arterial chemoembolisation (TACE) and systemic therapy.

The effect of BMI on immunotherapy has been evaluated in multiple cancer sites. Overweight/obese patients receiving immune checkpoint inhibition have favourable OS and PFS across multiple cancer sites [18, 19] including melanoma [20], renal cell carcinoma [18, 19] and non-small cell lung cancer [21] (NSCLC); the largest study being in 1434 patients with NSCLC demonstrating a survival benefit of atezolizumab in overweight/obese individuals [21]. The effect of BMI on bevacizumab response has similarly been evaluated in multiple cancer types, with positive [22], negative [23] and no associations [24] seen across multiple cancer sites. The association of BMI with immunotherapy in HCC is less well studied, with a single study demonstrating that BMI ≥ 25 is associated with improved OS in patients receiving PD-1 antibody-based regimens (17.5 vs. 5.0 months; *p* = 0.034). No difference in PFS was observed (2.7 vs. 2.9 months; *p* = 0.74) [25]. In this study, over 70% of patients had previous systemic treatment, and the effects of BMI on treatment-naïve patients receiving combination immunotherapy is not known.

To-date no study has evaluated the association of BMI on efficacy and safety outcomes of atezolizumab plus bevacizumab used for the first-line treatment of HCC in routine clinical practice. We conducted a retrospective analysis of patients receiving atezolizumab plus bevacizumab for unresectable HCC across seven tertiary centres, evaluating the effect of BMI on efficacy and safety.

## Methods and materials

### Study participants and design

Patients previously undergoing systemic therapy, including oral multikinase inhibitors and immune checkpoint inhibitors, were excluded from the study. Consecutive patients with unresectable HCC receiving atezolizumab plus bevacizumab across eight tertiary centres in Germany (*n* = 30), Japan (*n* = 51), Austria (*n* = 12), United Kingdom (*n* = 15), Italy (*n* = 12), Taiwan (*n* = 11) and United States of America (*n* = 60) were recruited in the study. Inclusion criteria included the following : age at least 18 years old; histological or radiological diagnosis of HCC in accordance with the American Association for the Study of Liver Diseases (AASLD) criteria [26]; diagnosis of advanced disease as per Barcelona Clinic Liver Cancer (BCLC) criteria [27]—BCLC C or BCLC B not amenable to locoregional therapy.

### Treatment protocol

Combination atezolizumab plus bevacizumab were administered according to the IMbrave150 protocol [5]: atezolizumab 1200 mg and bevacizumab 15 mg/kg intravenously every 3 weeks. Toxicity management and dose modifications were managed by local institutions as per summary of product characteristics (SmPC). Treatment was continued until disease progression or unacceptable toxicity as per local multidisciplinary assessment.

### Patient outcomes

Patients’ baseline demographics were collected retrospectively, and clinical outcomes were prospectively maintained at each participating site. Radiological response following atezolizumab plus bevacizumab therapy was assessed as per RECIST criteria v1.1 [28] on CT or MRI performed at 9–12 week intervals. Overall response rate was defined as all patients having complete response (CR) or partial response (PR). Disease control rate included all patients with CR, PR and stable disease (SD).Treatment-related adverse effects (trAEs) were assessed at every point of patient contact and graded as per the National Cancer Institute Common Terminology Criteria for Adverse Events (CTCAE) v. 5.0 [29]. Atezolizumab-related and bevacizumab-related adverse events were defined by the treating physician at each treatment centre as per the SmPC for each drug.

### Statistical analysis

For BMI analysis, patients were divided into two cohorts. BMI was defined as height (in metres) divided by weight squared (in kilograms). Patients were divided into those with a BMI of 25 or greater (overweight) and those with a BMI less than 25 (non-overweight). Baseline characteristics were compared within the divided BMI (overweight vs. non-overweight). *χ*^2^ test was used to compare categorical data, and unpaired student *t* test for continuous data. Treatment-related adverse events and ORR/DCR were compared between BMI cohorts using *χ*^2^ test. The distribution of BMI with patient characteristics were determined using Pearson’s correlation coefficient for continuous variables and unpaired student *t* tests for categorical variables.

Time-to-event analysis was performed using Kaplan–Meier method. OS was defined as the time in months from the date of first administration of atezolizumab plus bevacizumab to date of death or last follow-up. PFS was defined as time in months from the date of first treatment to date of death or date of progression on radiological imaging. OS and PFS were compared between overweight and non-overweight cohorts using log-rank. A *p* value of less than 0.05 was defined as statistically significant. Univariate and multivariate Cox regression models for overweight/non-overweight cohorts and established prognostic factors were conducted for OS and PFS. BCLC stage (C vs. A or B), Child-Turcotte-Pugh class (B vs. A), tumour size (7 cm vs. ≤ 7 cm), macrovascular invasion (MVI), metastatic disease and alpha-fetoprotein (AFP) (> 400 ng/dL vs. ≤ 400 ng/dL) are all prognostic factors previously shown to correlate with HCC survival [30] and were included in Cox regression models. We conducted further analysis assessing the impact of different BMI classes on patient survival. BMI classes were defined as underweight (BMI < 18.5), normal (18.5 ≤ BMI < 25), overweight (25 ≤ BMI < 30) and obese (BMI ≥ 30). Kaplan–Meier analysis and Cox regression models were performed for the different BMI classes.

## Results

### Baseline characteristics

191 patients received atezolizumab plus bevacizumab consecutively across eight tertiary referral centres. The baseline characteristics of patients are shown in Table 1. Viral hepatitis was the leading cause of chronic liver disease, with 23.0% of patients with Child-Turcotte-Pugh class B. Prior to immunotherapy, 60.1% of patients were BCLC stage C and 37.7% of patients had extra-hepatic disease. Correlation of BMI with patient characteristics is shown in Supplementary Fig. 1. Mean BMI was significantly elevated in patients with underlying non-alcoholic fatty liver disease (NAFLD) (28.7 vs. 25.3; *p* = 0.001). Patients with viral hepatitis had a lower BMI (24.9 vs. 26.7; *p* = 0.01). Ninety-four patients (49.2%) had a BMI of 25 or greater. Average BMI was 29.6 ± 3.4 vs. 21.9 ± 2.2 (*p* < 0.0001) in the overweight group compared to the non-overweight group. In the overweight group 43 (45.7%) patients were obese. A higher proportion of NAFLD was present in the overweight patients (19.2% vs. 7.2%; *p* = 0.01), with lower rates of chronic hepatitis B seen (12.8% vs. 25.8%; *p* = 0.02). There was a lower rate of extrahepatic disease in the overweight cohort (29.8% vs. 45.4%; *p* = 0.03) and a higher rate of macrovascular invasion in the overweight group compared to the non-overweight group (4.3% vs. 5.4%; *p* = 0.05). A lower proportion of overweight patients had a previous resection (14.9% vs. 30.9%; *p* = 0.02), with other previous treatments comparable between the two groups.

**Table 1:** Baseline characteristics of study population stratified by BMI

	All patients (*n* = 191)	BMI < 25 (*n* = 97)	BMI 25 + (*n* = 94)	*p* value
Centre				
Germany	30 (15.7)	12 (12.4)	18 (19.2)	0.06
Austria	121 (6.3)	3 (3.1)	9 (9.6)	
United Kingdom	15 (7.9)	6 (6.2)	9 (9.6)	
Italy	12 (6.3)	6 (6.2)	6 (6.4)	
United States of America	60 (31.4)	29 (29.9)	31 (33.0)	
Japan	51 (26.7)	35 (36.1)	16 (17.0)	
Taiwan	11 (5.8)	6 (6.2)	5 (5.3)	
Median Age (IQR)	68.4 (61.8–75.2)	68.2 (60.4–75.2)	69.3 (62.2–75.0)	0.76
Male Sex	161 (84.3)	83 (85.6)	78 (83.0)	0.78
Risk factors for chronic liver disease				
Non-alcoholic fatty liver disease	25 (13.1)	7 (7.2)	18 (19.2)	0.01
Alcohol related	73 (38.2)	36 (37.1)	37 (39.4)	0.75
Hepatitis B infection	37 (19.4)	25 (25.8)	12 (12.8)	0.02
Hepatitis C infection	72 (37.7)	40 (41.2)	32 (34.0)	0.31
Other	12 (8.6)	6 (9.7)	6 (7.7)	0.68
Child-Turcotte-Pugh class				
A	147 (77.0)	77 (79.4)	70 (74.5)	0.42
B	44 (23.0)	20 (20.6)	24 (25.5)	
Varices present	39 (20.4)	15 (15.5)	24 (25.5)	0.08
Maximum tumor diameter (cm)	6.8 (4.9)	6.8 (5.4)	6.9 (4.3)	0.92
Macrovascular invasion (MVI)	78 (40.8)	33 (34.0)	45 (47.9)	0.05
AFP (ng/dL)				
≤ 400	126 (66.0)	68 (66.0)	58 (61.7)	0.22
> 400	65 (34.0)	33 (34.0)	36 (38.3)	
Extrahepatic spread (EHS)	72 (37.7)	44 (45.4)	28 (29.8)	0.03
ECOG-PS				
0	119 (63.0)	64 (66.7)	55 (59.1)	0.09
1	64 (33.9)	27 (28.1)	37 (39.8)	
2	6 (3.2)	5 (5.2)	1 (1.1)	
Barcelona clinic liver cancer stage				
A	7 (3.7)	4 (4.2)	3 (3.3)	0.24
B	68 (36.2)	40 (41.7)	28 (30.4)	
C	113 (60.1)	52 (54.2)	61 (66.3)	
Previous locoregional treatment				
Resection	44 (23.0)	30 (30.9)	14 (14.9)	0.02
Radiofrequency ablation	38 (19.9)	20 (20.6)	18 (19.2)	0.80
Transarterial chemoembolization	57 (29.8)	31 (32.0)	26 (27.7)	0.52
Y90	21 (11.0)	7 (7.2)	14 (14.9)	0.09
External Beam Radiotherapy	6 (3.1)	4 (4.1)	2 (2.1)	0.43

*n* (%) for discrete variables; mean ± standard deviation for continuous variables

*AFP* alpha-fetoprotein, *ECOG-PS* Eastern cooperative oncology group performance status

### Efficacy

**Fig. 1:**
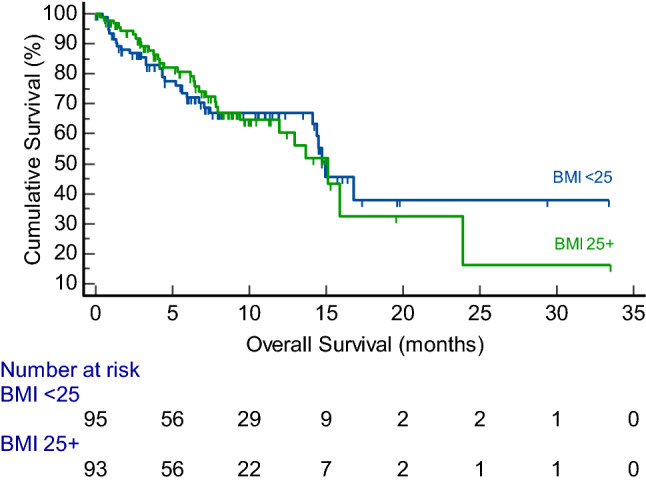
Kaplan–Meier curve showing overall survival (months) for overweight (BMI ≥ 25) and non-overweight (BMI < 25) patients with unresectable hepatocellular carcinoma patients after atezolizumab plus bevacizumab administration

**Fig. 2:**
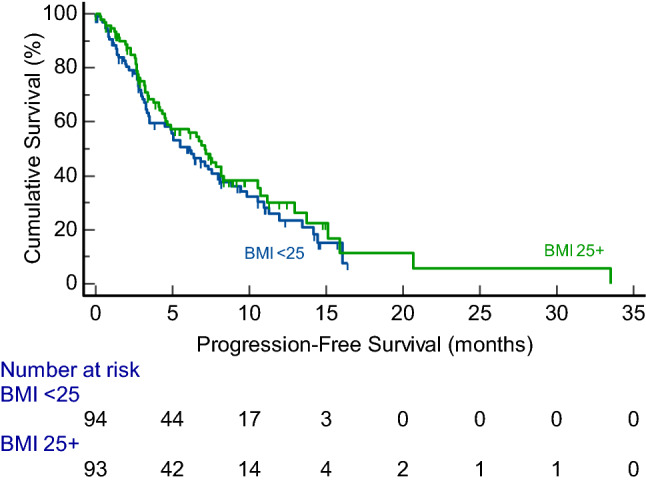
Kaplan–Meier curve showing progression-free survival (months) for overweight (BMI ≥ 25) and non-overweight (BMI < 25) patients with unresectable hepatocellular carcinoma patients after atezolizumab plus bevacizumab administration

Median overall survival for the entire cohort was 14.9 months (95% CI 13.6–23.9 months). Seventy-four patients died during the study observation period. Median progression-free survival was 6.7 months (95% CI 4.9–7.9 months). Overweight patients had a similar survival compared to non-overweight patients (median OS: 15.1 vs. 14.9 months; *p* = 0.99) (Fig. 1). Similarly, median PFS was comparable between the overweight and non-overweight cohorts (median PFS: 7.1 vs. 6.1 months; *p* = 0.42) (Fig. 2). Overweight BMI did not impact overall survival in univariate (HR 1.00, 95% CI 0.60–1.64); *p* = 0.98) and multivariate (HR 0.72, 95% CI 0.42–1.23); *p* = 0.23) analysis (Table 2). Similarly, no effect was observed for progression-free survival (HR 0.70, 95% CI 0.41–1.20; *p* = 0.20) (Table 3).

**Table 2:** Effects of BMI and prognostic factors on overall survival after atezolizumab and bevacizumab in univariate and multivariate Cox regression models

	Univariate models		Multivariable models	
	Hazard ratio (95% CI)	*p* value	Hazard ratio (95% CI)	*p* value
BMI 25+	1.00 (0.60–1.64)	0.98	0.72 (0.42–1.23)	0.23
BCLC stage (C vs A or B)	1.50 (0.89–2.52)	0.13	1.06 (0.57–1.97)	0.85
CTP class (B vs A)	3.01 (1.77–5.13)	< 0.001	2.44 (1.35–4.42)	0.003
Tumour size > 7 cm	1.30 (0.77–2.20)	0.32	1.04 (0.61–1.78)	0.89
MVI	2.51 (1.15–4.18)	< 0.001	1.87 (0.99–3.55)	0.05
Metastatic disease	0.80 (0.47–1.36)	0.41	0.88 (0.49–1.57)	0.66
AFP > 400 ng/dL	1.32 (0.79–2.19)	0.29	1.21 (0.71–2.05)	0.49

*95% CI* 95% confidence interval, *BCLC* Barcelona clinic liver cancer, *CTP* Child-Turcotte-Pugh, *MVI* Macrovascular invasion, *AFP* alpha-fetoprotein

**Table 3:** Effects of BMI and prognostic factors on progression-free survival after atezolizumab and bevacizumab in univariate and multivariate Cox regression models

	Univariate models		Multivariable models	
	Hazard ratio (95% CI)	*p* value	Hazard ratio (95% CI)	*p* value
BMI 25 +	0.90 (0.55–1.49)	0.69	0.70 (0.41–1.20)	0.20
BCLC stage (C vs A or B)	1.56 (0.92–2.64)	0.10	1.03 (0.54–1.94)	0.94
CTP class (B vs A)	2.29 (1.35–3.87)	0.002	1.88 (1.04–3.40)	0.04
Tumour size > 7 cm	1.28 (0.76–2.15)	0.36	1.04 (0.61–1.79)	0.88
MVI	2.30 (1.38–3.82)	0.001	1.92 (0.99–3.73)	0.06
Metastatic disease	1.03 (0.60–1.75)	0.92	1.11 (0.62–1.98)	0.74
AFP > 400 ng/dL	1.35 (0.81–2.26)	0.24	1.19 (0.69–2.04)	0.53

*95% CI* 95% confidence interval, *BCLC* Barcelona clinic liver cancer, *CTP* Child-Turcotte-Pugh, *MVI* Macrovascular invasion, *AFP* alpha-fetoprotein

Treatment response using the RECIST criteria was available in 163 patients. ORR and DCR for the entire cohort were 24.5% and 73.0%, respectively (Supplementary Table 1). Progressive disease was present in 27.0% of patients. Overweight patients had similar ORR (27.2% vs. 22.0%; *p* = 0.44) and DCR (74.1% vs. 71.9%;* p* = 0.76) compared to non-overweight patients.

We further assessed the impact of different BMI classes on survival after therapy. Baseline characteristics were comparable between the classes (Supplementary Table 2). There was no difference in median OS (underweight 11.4 vs. normal 18.8 vs. overweight 19.2 vs. obese 11.5; *p* = 0.67) and PFS (underweight 7.4 vs. normal 7.4 vs. overweight 9.9 vs. obese 8.7; *p* = 0.83) between BMI classes (Supplementary Figs. 2 and 3). There was no impact of BMI class on survival in univariate and multivariate Cox regression models (Supplementary Tables 3 and 4).

The difference in rates of MVI and extrahepatic spread observed in the overweight and non-overweight groups may impact survival outcomes. We conducted subgroup analysis assessing survival in overweight and non-overweight patients without MVI or extrahepatic spread. Overweight and non-overweight patients had comparable OS (median OS 23.8 vs. 14.9 months; *p* = 0.26) and PFS (median PFS 20.7 vs. 14.2 months; *p* = 0.27). Similarly, overweight BMI did not impact OS or PFS in patients with MVI and/or extrahepatic spread.

### Safety

In total, 127 patients experienced treatment-related adverse events. Similar number of patients experienced atezolizumab-related (43.5%) and bevacizumab-related (43.5%) adverse events (Table 4). Thirty-nine patients (20.4%) experience at least one grade 3 or greater adverse event. Twelve patients (6.3%) discontinued combination therapy due to treatment-related adverse events. Atezolizumab-related fatigue was higher in the overweight group compared to the non-overweight group (22.3% vs. 10.3%; *p* = 0.02). Atezolizumab-related thyroid dysfunction was lower in the overweight group compared to the non-overweight group (1.1% vs. 8.3%; *p* = 0.02). Bevacizumab-related thrombosis was higher in overweight patients (8.5% vs. 2.1%; *p* = 0.045). However, trAEs requiring drug discontinuation were similar between the two cohorts (7.4% vs. 5.2%; *p* = 0.51). When reviewing BMI class, atezolizumab-related thyroid dysfunction was significantly higher in underweight (20.0%) and normal BMI (6.9%) patients compared to overweight and obese patients (Supplementary Table 5). Rates of bevacizumab-related thrombosis were significantly higher in obese patients (14.0%).

**Table 4:** Atezolizumab and bevacizumab treatment-related adverse events stratified by BMI

	All patients (*n* = 191)	BMI < 25 (*n* = 97)	BMI 25+ (*n* = 94)	*p* value
Any grade trAEs	127 (66.5)	62 (63.9)	65 (69.2)	0.44
Grade ≥ 3^a^ trAEs	39 (20.4)	21 (21.7)	18 (19.2)	0.67
Atezolizumab-related	15 (7.9)	11 (11.3)	4 (4.3)	0.07
Bevacizumab-related	26 (13.6)	12 (12.4)	14 (14.9)	0.61
trAEs requiring drug discontinuation	12 (6.3)	5 (5.2)	7 (7.4)	0.51
Atezolizumab trAEs				
Overall	83 (43.5)	42 (43.3)	41 (43.6)	0.96
Fatigue	31 (16.2)	10 (10.3)	21 (22.3)	0.02
Hepatotoxicity	28 (14.7)	16 (16.5)	12 (12.8)	0.47
Skin toxicity	9 (4.7)	3 (3.1)	6 (6.4)	0.28
Colitis	24 (12.6)	12 (12.4)	12 (12.8)	0.93
Thyroid dysfunction	9 (4.7)	8 (8.3)	1 (1.1)	0.02
Pneumonitis	4 (2.1)	1 (1.0)	3 (3.2)	0.30
Bevacizumab trAEs				
Overall	83 (43.5)	40 (41.2)	43 (45.7)	0.53
Bleeding	20 (10.5)	10 (10.3)	10 (10.6)	0.94
Hypertension	44 (23.0)	23 (23.7)	21 (22.3)	0.82
Proteinuria	38 (19.9)	23 (23.7)	15 (16.0)	0.18
Thrombosis	10 (5.2)	2 (2.1)	8 (8.5)	0.045

^a^graded as per the National Cancer Institute Common Terminology Criteria for Adverse Events (CTCAE)

*trAE* treatment-related adverse event

## Discussion

This multi-centre study is the first to evaluate the effect of BMI on treatment efficacy and safety of Atezo/Bev for advanced/unresectable HCC. We show a BMI of 25 and above is associated with similar OS and PFS compared to patients with normal or underweight BMI. We observe higher rates of atezolizumab-related fatigue and bevacizumab-related thrombosis in overweight patients, without an increase in overall trAEs or treatment discontinuation.

Studies evaluating the effect of BMI on HCC survival have demonstrated varied results. Elevated BMI does not influence survival in patients undergoing curative resection [16, 17] and TACE [15]. In contrast, a positive association between BMI and survival for patients undergoing systemic chemotherapy has been reported [14, 31]. Secondary analysis of a phase III study demonstrated a BMI 25 or above was associated with an increased OS but not PFS in 544 patients receiving sorafenib for unresectable HCC [14]. These findings were replicated in a Japanese cohort of 234 patients receiving sorafenib [31]. However, there is a paucity of studies investigating the impact of BMI on outcomes secondary to immunotherapy in HCC. In a large meta-analysis of 4900 patients from 16 studies that investigated the prognostic role of BMI in patients receiving immunotherapy for different cancer sites (not including HCC) [18], overweight/obese patients showed reduced mortality compared to normal/underweight BMI patients in advanced melanoma (HR 0.69; 95% CI 0.51–0.95), NSCLC (HR 0.82; 95% CI 0.66–1.01) but not renal cell or other cancer types. High BMI was associated with improved PFS across all cancer types (HR 0.87; 95% CI 0.48–0.95), though not replicated in single cancer-site analysis. The authors reported that adverse events, assessed from four studies, were higher in the overweight/obese cohort. A retrospective study specifically evaluating anti-PD-1/PD-L1 in NSCLC, melanoma and renal cell carcinoma patients also showed OS, PFS and ORR were significantly longer in overweight/obese patients [19]. We did not observe this increased survival overweight patients with HCC. There may be several reasons for this observation. In the meta-analysis, only 35 (3.6%) patients underwent PD-L1 inhibition with atezolizumab, with over 96% of patients receiving PD-1 inhibitors nivolumab and pembrolizumab. Inter-class variation in efficacy of immunotherapy agents has been previously reported [32], and elevated BMI may affect anti-PD-1 and anti-PD-L1 response differentially. Additionally, we observed higher rates of NAFLD in patients with the overweight cohort. Previous studies have demonstrated a lack of efficacy of immunotherapy in patients with non-viral HCC [6, 33, 34]. This may be due to aberrant T-cell activation within hepatic tissue impairing immune response to checkpoint inhibition [33]. Therefore, underlying NAFLD and changes in the hepatic tissue immune environment in overweight HCC patients may attenuate the survival benefit from immunotherapy observed in other cancer sites.

Only a single previous study has evaluated effect of BMI on response to immunotherapy in unresectable HCC [25]. The effect of BMI and sarcopenia was investigated in 57 patients receiving anti-PD-1 antibody combination therapy in a single centre. Median OS was significantly longer in the BMI ≥ 25 group compared to BMI < 25 (17.5 vs. 5.0 months; *p* = 0.034), with similar PFS (2.7 vs. 2.9 months). The author’s observed sarcopenia was associated with a non-significant reduced OS (5.0 vs. 14.3 months; *p* = 0.054), which may correlate with lower BMI. The majority of patients in this study had received previous lines of systemic therapy, and 41% had a PS of 2–3, factors which may have adversely impacted on both BMI and sarcopenia, as it is likely that patients with poor PS will have a degree of cachexia [35]. Furthermore, no information is given by the authors regarding the type of immunotherapy administered. We did not observe a correlation between BMI and either OS or PFS in our larger cohort of patients receiving first-line treatment with anti-PD-L1 antibody and anti-VEGF therapy. The differences observed between the studies may be attributed to mechanistic differences of pharmacotherapy and differences in patient demographics as described.

Underlying NAFLD is highly represented in our cohort of patients with elevated BMI. This is expected as elevated BMI is associated with NAFLD as part of the metabolic syndrome spectrum [36]. The efficacy of immunotherapy in patients with NAFLD remains debated. A subgroup analysis of IMbrave150 showed no survival benefit with Atezo/Bev compared to sorafenib in patients with non-viral HCC [6]. This lack of efficacy of immunotherapy in non-viral related HCC was further demonstrated in two meta-analyses [33, 34]. However, these studies did not distinguish between alcohol-related and NAFLD-related HCC. We observe a higher rate of NAFLD-related HCC in our overweight cohort, with no difference in survival compared to the non-overweight group. These results suggest Atezo/Bev is efficacious in NAFLD patients, but further prospective study is needed. We found a higher rate of macrovascular invasion with overweight patients, which has been shown to be associated with NAFLD [37] as have larger sized tumors [38]. The higher rates of MVI in overweight patients may arise due to the effect of adipose tissue on the tumors microenvironment. Increased adipose tissue is associated with higher rates of hypoxia, causing release of pro-inflammatory cytokines including monocyte chemoattractant protein-1, interleukin-1β and tumors necrosis factor-α [34, 35]. Higher levels of Interleukin-6 are seen in obesity, with increased secretion from hepatic stellate cells and Kupffer cells within the liver [41]. Cytokine release drives chronic inflammation through macrophage and lymphocyte infiltration [42], promoting angiogenesis and alterations in the extracellular matrix leading to tumors growth [43]. In liver tissue, excessive saturated fatty acids can result in alterations in glucose metabolism and production of reactive oxidative species leading to progression of hepatocellular carcinoma [44, 45]. Diabetes mellitus is associated with higher rates of MVI in HCC patients [46], consistent with our observations with overweight and NAFLD patients, as part of the metabolic syndrome spectrum. Though the higher rates of MVI in the elevated BMI cohort did impact survival after immunotherapy, it may have implications for other treatments in overweight patients, including curative therapy such as hepatectomy and liver transplantation.

The impact of BMI on the safety of immunotherapy in patients has been evaluated. Cortellini et al., showed higher BMI was associated with higher rates of trAEs and subsequent treatment discontinuation in a cohort of 1070 patients receiving PD-1/PD-L1 inhibition for multiple primary cancer sites [47]. The authors observed BMI as an independent predictor for trAE in multivariate analysis. The authors speculate the higher rate of trAEs observed with higher efficacy may represent an immunogenic phenotype observed in higher BMI patients [48]. Similarly, higher BMI was associated with increase in overall trAEs in a meta-analysis of 4090 patients across multiple cancer sites receiving immunotherapy [18]. In our study, we did not report an overall increase in overall trAEs in overweight patients. We observed higher rates of atezolizumab-related fatigue and bevacizumab-related thrombosis in the overweight cohort. The mechanism for bevacizumab-related thrombosis is unclear, though may be mediated by increased vascular damage and inflammation [49]. Elevated BMI is an established risk factor for thrombosis through promotion of chronic inflammation and impaired fibrinolysis [50]. This is consistent with the higher rates of macrovascular invasion, encompassing portal vein thrombosis, observed in our overweight cohort. Sparks et al., observed BMI did not impact on rates of thrombosis in colorectal, ovarian, lung and gliblastoma multiforme cancer patients receiving bevacizumab [51]. The higher rates of thrombosis observed in our overweight cohort of HCC patients may be due to underlying liver dysfunction. Patients with cirrhosis are at increased risk of both bleeding and thrombosis [52], due to the liver’s role in synthesising both anticoagulant and coagulant factors. Obesity is an independent risk factor for thrombosis in pre-transplant cirrhosis patients [53] and may be a key driver in the increased risk of bevacizumab-related thrombosis in HCC patients. Atezolizumab-related thyroiditis has been seen in up to 10% of cases [54]. We observe a lower rate of atezolizumab-related thyroid dysfunction in the overweight cohort. The relationship between elevated BMI and thyroid dysfunction is complex, and mediated by adipocytes, cytokines and iodine uptake in thyroid cells [55]. This interplay may influence the rates of immunotherapy-related thyroid dysfunction in overweight patients. The difference in trAEs observed between the overweight and non-overweight cohorts may also be due to unmeasured confounding factors between the two groups. Further large studies will be required to assess trAEs with increased use of atezolizumab plus bevacizumab specific for HCC.

Our study has limitations. Though follow-up was prospectively collected, this is a retrospective study which is subject to selection and collection bias. Baseline characteristics influencing survival such as underlying NAFLD and extrahepatic spread were higher in the overweight cohorts and may have an impact on patient survival. Additionally, there may be unmeasured confounding factors. Though patients received treatment from tertiary centres, inter-site variation in treatment protocols, follow-up and efficacy and safety assessments cannot be excluded. Lower BMI may represent poor global nutritional status and sarcopenia. Clinically and radiologically measured sarcopenia is associated with increased mortality in patients with cirrhosis [56] and HCC [57] and, therefore, may confound our findings. Further studies assessing sarcopenia and other body composition measures such as subcutaneous adipose tissue, visceral adipose tissue and muscle volume would allow understanding of the impact of muscle mass and adipose tissue on immunotherapy response in HCC. In survival analysis we report all-cause mortality rather than liver-specific mortality. This may be influenced by unreported medical comorbidities. Despite these limitations, this study represents the largest cohort of post-registration real-time use of atezolizumab plus bevacizumab for HCC in overweight patients. As an increasing number of immunotherapy agents emerge for use in HCC [58], further studies evaluating the efficacy and safety of these agents in overweight patients will guide future clinical practice.

## Conclusion

Our study demonstrates atezolizumab plus bevacizumab therapy is associated with comparable efficacy in overweight HCC patients with higher rates of NALFD. Increased body mass index is associated with higher rates of treatment-related fatigue and thrombosis, but no increase in overall treatment-related adverse events. Combination therapy is safe and efficacious to use in overweight patients, including those with underlying NAFLD.

## Supplementary Information

Below is the link to the electronic supplementary material.

Supplementary file1 (PDF 865 KB)

## Data Availability

All data is available upon request from the corresponding author Dr Rohini Sharma, email: r.sharma@imperial.ac.uk.

## Abbreviations

HCC: Hepatocellular carcinoma
PD-L1: Programmed death ligand 1
VEGF: Vascular endothelial growth factor
Atezo/Bev: Atezolizumab and bevacizumab
OS: Overall survival
PFS: Progression-free survival
HR: Hazard ratio
CI: Confidence interval
STRIDE: Single tremelimumab regular interval durvalumab
BMI: Body mass index
TACE: Trans-arterial chemoembolization
NSCLC: Non-small cell lung cancer
AASLD: American Association for the Study of Liver Diseases
BCLC: Barcelona clinic liver cancer
SmPC: Summary of product characteristics
CR: Complete response
PR: Partial response
SD: Stable disease
trAE: Treatment-related adverse event
CTCAE: Common terminology criteria for adverse events
MVI: Macrovascular invasion
AFP: Alpha-fetoprotein
NAFLD: Non-alcoholic fatty liver disease
