# Supplementary material for: Impact of body mass index in patients receiving atezolizumab plus bevacizumab for hepatocellular carcinoma
Source: Hepatol Int. 2023 Apr 1;17(4):904–14. doi: 10.1007/s12072-023-10491-3 (PMC10386929; doi:10.1007/s12072-023-10491-3)
Supplement: Supplementary file 1 — Supplementary file1 (PDF 865 KB) [file 12072_2023_10491_MOESM1_ESM.pdf]

Impact of body mass index in patients receiving atezolizumab plus bevacizumab for hepatocellular carcinoma

Mathew Vithayathil[1], Antonio D'Alessio[1,2], Claudia Angela Maria Fulgenzi[1,3], Naoshi Nishida[4], Martin Schönlein[5], Johann von Felden[6], Kornelius Schulze[6], Henning Wege[6], Anwaar Saeed[7], Brooke Wietharn[7], Hannah Hildebrand[7], Linda Wu[8], Celina Ang[8], Thomas U Marron[8], Arndt Weinmann[9], Peter R Galle[9], Dominik Bettinger[10], Bertram Bengsch[10,11,12], Arndt Vogel[13], Lorenz Balcar[14], Bernhard Scheiner[14], Pei-Chang Lee[15], Yi-Hsiang Huang[15,16], Suneetha Amara[17], Mahvish Muzaffar[17], Abdul Rafeh Naqash[17,18], Antonella Cammarota[2,19], Valentina Zanuso[2,19], Tiziana Pressiani[19], Matthias Pinter[14], Alessio Cortellini[1], Masatoshi Kudo[4], Lorenza Rimassa[2,19], David J Pinato[1,20], Rohini Sharma[1]

1. Department of Surgery & Cancer, Imperial College London, London, UK.
2. Department of Biomedical Sciences, Humanitas University, Pieve Emanuele, Milan, Italy.
3. Division of Medical Oncology, Policlinico Universitario Campus Bio-Medico, Rome, Italy.
4. Department of Gastroenterology and Hepatology, Kindai University Faculty of Medicine, Osaka, Japan.
5. Department of Oncology, Hematology and Bone Marrow Transplantation with Section of Pneumology, University Medical Center Hamburg-Eppendorf, Hamburg, Germany.
6. Department of Gastroenterology and Hepatology, University Medical Center Hamburg-Eppendorf, Hamburg, Germany.
7. Division of Medical Oncology, Department of Medicine, Kansas University Cancer Center, Kansas City, Kansas, USA.
8. Division of Hematology/Oncology, Department of Medicine, Tisch Cancer Institute, Mount Sinai Hospital, New York, New York, USA.
9. I. Medical Department, University Medical Center Mainz, Mainz, Germany.

10. Department of Medicine II (Gastroenterology, Hepatology, Endocrinology and Infectious Diseases), Faculty of Medicine, Freiburg University Medical Center, University of Freiburg, Freiburg, Germany.
11. University of Freiburg, Signalling Research Centers BIOSS and CIBSS, Freiburg, Germany.
12. German Cancer Consortium (DKTK), partner site, Freiburg, Germany.
13. Hannover Medical School, Hannover, Germany.
14. Division of Gastroenterology & Hepatology, Department of Internal Medicine III, Medical University of Vienna, Vienna, Austria.
15. Division of Gastroenterology and Hepatology, Department of Medicine, Taipei Veterans General Hospital, Taipei, Taiwan.
16. Institute of Clinical Medicine, School of Medicine, National Yang Ming Chiao Tung University, Taipei, Taiwan.
17. Division of Hematology/Oncology, East Carolina University, Greenville, North Carolina, USA.
18. Medical Oncology/TSET Phase 1 Program, Stephenson Cancer Center, University of Oklahoma, Norman, Oklahoma, USA.
19. Medical Oncology and Hematology Unit, Humanitas Cancer Center, IRCCS Humanitas Research Hospital, Rozzano, Milan, Italy.
20. Division of Oncology, Department of Translational Medicine, University of Piemonte Orientale, Novara, Italy.

Author for correspondence

Dr Rohini Sharma, MBBS, PhD, FRACP, FRCP

Reader and Consultant in Medical Oncology and Clinical Pharmacology

Imperial College London Hammersmith Campus, Du Cane Road, W12 0HS, London (UK)

Tel: +44 020 3313 3059 E-mail: r.sharma@imperial.ac.uk

Supplementary Table 1. Best radiological response evaluated per RECIST criteria version 1.1 stratified by BMI..4

Supplementary Table 2. Baseline characteristics of study population stratified by BMI class.............5

Supplementary Table 3. Effects of BMI class and prognostic factors on overall survival after atezolizumab and bevacizumab in univariate and multivariate Cox regression models.......................7

Supplementary Table 4. Effects of BMI class and prognostic factors on progression-free survival after atezolizumab and bevacizumab in univariate and multivariate Cox regression models.......................8

Supplementary Table 5. Atezolizumab and bevacizumab treatment-related adverse events stratified by BMI class...9

Supplementary Figure 1. BMI association with baseline patient characteristics. *p* values shown for Pearson correlation coefficient for continuous variables and unpaired student t-test for categorical variables. Mean BMI (square) with 95% confidence interval (green) shown for categorical variables. 11

....11

Supplementary Figure 2. Kaplan-Meier curve showing overall survival (months) for underweight (BMI < 18.5), normal (18.5 ≤ BMI < 25), overweight (25 ≤ BMI < 30) and obese (BMI ≥ 30) patients with unresectable hepatocellular carcinoma patients after atezolizumab plus bevacizumab administration...12

Supplementary Figure 3. Kaplan-Meier curve showing progression-free survival (months) for underweight (BMI < 18.5), normal (18.5 ≤ BMI < 25), overweight (25 ≤ BMI < 30) and obese (BMI ≥ 30) patients with unresectable hepatocellular carcinoma patients after atezolizumab plus bevacizumab administration...13

Supplementary Table 1. Best radiological response evaluated per RECIST criteria version 1.1 stratified by BMI

	**All patients** (n=163[a])	**BMI <25** (n=64)	**BMI 25+** (n=59)	***p value***
Complete Response	0	0	0	
Partial Response	40 (24.5)	18 (22.0)	22 (27.2)	0.44
Stable Disease	79 (48.5)	41 (50.0)	38 (46.9)	0.69
Progressive Disease Rate	44 (27.0)	23 (28.1)	21 (25.9)	0.76

Notes: [a]Radiological response was assessed in 163 patients (85.3%);

Abbreviations: RECIST, response evaluation criteria in solid tumours.

Supplementary Table 2. Baseline characteristics of study population stratified by BMI class.

	Underweight (n=10)	Normal (n=87)	Overweight (n=51)	Obese (n=43)	*p value*
**Centre**					
Germany	3 (30.0)	9 (10.3)	9 (17.7)	9 (20.9)	
Austria	1 (10.0)	2 (2.3)	4 (7.8)	5 (11.6)	
United Kingdom	0	6 (6.9)	4 (7.8)	5 (11.6)	
Italy	0	6 (6.9)	4 (7.8)	2 (4.7)	0.02
United States of America	2 (20.0)	27 (31.0)	11 (21.6)	20 (46.5)	
Japan	4 (40.0)	31 (35.6)	14 (27.5)	2 (4.7)	
Taiwan	0	6 (6.9)	5 (9.8)	0	
**Median Age (IQR)**	69.7 (63.0-77.3)	68.2 (59.9-75.2)	70.6 (64.1-76.0)	65.5 (59.5-74.6)	0.26
**Male Sex**	8 (80.0)	75 (86.2)	43 (84.3)	35 (81.4)	0.98
**Risk factors for chronic liver disease**					
Non-alcoholic fatty liver disease	1 (10.0)	6 (6.9)	8 (15.7)	10 (23.3)	0.07
Alcohol related	4 (40.0)	32 (36.8)	15 (29.4)	22 (51.2)	0.19
Hepatitis B infection	0	25 (28.7)	10 (19.6)	2 (4.7)	0.004
Hepatitis C infection	5 (50.0)	35 (40.2)	14 (27.5)	18 (41.9)	0.32
Other	2 (33.3)	4 (7.1)	3 (8.1)	3 (7.3)	0.18
**Child-Turcotte-Pugh class**					
A	8 (80.0)	69 (79.3)	42 (82.4)	28 (65.1)	0.21
B	2 (20.0)	18 (20.7)	9 (17.7)	15 (34.9)	0.21
**Varices present**	1 (10.0)	14 (16.1)	14 (27.5)	10 (23.3)	0.33
**Maximum Tumor Diameter (cm)**	7.3 (6.2)	6.8 (5.3)	6.6 (4.7)	7.2 (3.8)	0.92

**Macrovascular invasion (MVI)**	1 (10.0)	32 (36.8)	22 (43.1)	23 (53.5)	0.06
**AFP (ng/dL)**					
≤400	9 (90.0)	59 (67.8)	31 (60.8)	27 (62.8)	0.32
>400	1 (10.0)	28 (32.2)	20 (39.2)	16 (37.2)	
**Extrahepatic spread (EHS)**	4 (40.0)	40 (46.0)	18 (35.3)	10 (23.3)	0.09
**ECOG-PS**					
0	6 (60.0)	58 (67.4)	33 (64.7)	22 (52.4)	
1	4 (40.0)	23 (26.7)	18 (35.3)	19 (45.2)	0.24
2	0	5 (5.8)	0	1 (2.4)	
**Barcelona Clinic Liver Cancer Stage**					
A	0	4 (4.7)	1 (2.0)	2 (4.8)	
B	5 (50.0)	35 (40.7)	19 (38.0)	9 (21.4)	0.36
C	5 (50.0)	47 (54.7)	30 (60.0)	31 (73.8)	
**Previous locoregional treatment**					
Resection	3 (30.0)	27 (31.0)	9 (17.7)	5 (11.6)	0.15
Radiofrequency ablation	3 (30.0)	17 (19.5)	8 (15.7)	10 (23.3)	0.68
Transarterial chemoembolization	4 (40.0)	27 (31.0)	14 (27.5)	12 (27.9)	0.86
Y90	1 (10.0)	6 (6.9)	8 (15.7)	6 (14.0)	0.39
External Beam Radiotherapy	0	4 (4.6)	2 (3.9)	0	0.49

Notes: n (%) for discrete variables; mean ± standard deviation for continuous variables

Abbreviations: AFP – alpha-fetoprotein; ECOG-PS – Eastern Cooperative Oncology Group Performance Status.

Supplementary Table 3. Effects of BMI class and prognostic factors on overall survival after atezolizumab and bevacizumab in univariate and multivariate Cox regression models

	Univariate models		Multivariable models	
	Hazard Ratio (95% CI)	*p* value	Hazard Ratio (95% CI)	*p* value
**BMI Class**				
Underweight vs Normal	1.41 (0.49-4.06)	0.52	2.19 (0.72-6.61)	0.17
Overweight vs Normal	0.84 (0.44-1.60)	0.60	0.73 (0.38-1.40)	0.34
Obese vs Normal	1.29 (0.70-2.39)	0.42	0.83 (0.42-1.64)	0.59
**BCLC Stage (C vs A or B)**	1.50 (0.89-2.52)	0.13	1.00 (0.54-1.86)	0.99
**CTP Class (B vs A)**	3.01 (1.77-5.13)	<0.0001	2.42 (1.32-4.45)	0.004
**Tumour size > 7cm**	1.30 (0.77-2.20)	0.32	1.06 (0.62-1.81)	0.84
**MVI**	2.51 (1.51-4.18)	<0.0001	2.04 (1.05-3.94)	0.03
**Metastatic Disease**	0.80 (0.47-1.36)	0.41	0.91 (0.51-1.63)	0.75
**AFP > 400 ng/dL**	1.32 (0.79-2.19)	0.29	1.21 (0.72-2.06)	0.47

Abbreviations: 95% CI – 95% Confidence Interval; BCLC – Barcelona Clinic Liver Cancer; CTP – Child-Turcotte-Pugh; MVI – Macrovascular invasion; AFP – alpha-fetoprotein;

Supplementary Table 4. Effects of BMI class and prognostic factors on progression-free survival after atezolizumab and bevacizumab in univariate and multivariate Cox regression models

	Univariate models		Multivariable models	
	Hazard Ratio (95% CI)	*p* value	Hazard Ratio (95% CI)	*p* value
**BMI Class**				
Underweight vs Normal	1.23 (0.43-3.51)	0.70	1.84 (0.61-5.60)	0.28
Overweight vs Normal	0.76 (0.39-1.47)	0.42	0.69 (0.36-1.34)	0.27
Obese vs Normal	1.11 (0.60-2.06)	0.73	0.80 (0.41-1.56)	0.80
**BCLC Stage (C vs A or B)**	1.56 (0.92-2.64)	0.10	0.96 (0.51-1.82)	0.91
**CTP Class (B vs A)**	2.29 (1.35-3.87)	0.002	1.85 (1.01-3.39)	0.045
**Tumour size > 7cm**	1.28 (0.76-2.15)	0.36	1.04 (0.61-1.79)	0.88
**MVI**	2.30 (1.38-3.82)	0.001	2.07 (1.04-4.13)	0.04
**Metastatic Disease**	1.03 (0.60-1.75)	0.92	1.12 (0.62-2.03)	0.71
**AFP > 400 ng/dL**	1.35 (0.81-2.26)	0.24	1.22 (0.71-2.10)	0.47

Abbreviations: 95% CI – 95% Confidence Interval; BCLC – Barcelona Clinic Liver Cancer; CTP – Child-Turcotte-Pugh; MVI – Macrovascular invasion; AFP – alpha-fetoprotein;

Supplementary Table 5. Atezolizumab and bevacizumab treatment-related adverse events stratified by BMI class.

	**Underweight** (n=10)	**Normal** (n=87)	**Overweight** (n=51)	**Obese** (n=43)	***p value***
**Any grade trAEs**	7 (70.0	55 (63.2)	32 (62.8)	33 (76.7)	0.42
**Grade≥3[a] trAEs**	2 (20.0)	19 (21.8)	10 (19.6)	8 (18.6)	0.97
Atezolizumab-related	2 (20.0)	9 (10.3)	2 (3.9)	2 (4.7)	0.21
Bevacizumab-related	1 (10.0)	11 (12.6)	8 (15.7)	6 (14.0)	0.95
**trAEs requiring drug discontinuation**	1 (10.0)	4 (4.6)	3 (5.9)	4 (9.3)	0.72
**Atezolizumab trAEs**					
Overall	4 (40.0)	38 (43.7)	19 (37.3)	33 (51.2)	0.60
Fatigue	2 (20.0)	8 (9.2)	10 (19.6)	11 (25.6)	0.09
Hepatotoxicity	1 (10.0)	15 (17.2)	8 (15.7)	4 (9.3)	0.64
Skin toxicity	1 (10.0)	2 (2.3)	3 (5.9)	3 (7.0)	0.49
Colitis	1 (10.0)	11 (12.6)	3 (5.9)	9 (20.9)	0.18
Thyroid dysfunction	2 (20.0)	6 (6.9)	1 (2.0)	0	0.02
Pneumonitis	0	1 (1.2)	1 (2.0)	2 (4.7)	0.58
**Bevacizumab trAEs**					
Overall	7 (70.0)	33 (37.9)	23 (45.1)	20 (46.5)	0.24
Bleeding	2 (20.0)	8 (9.2)	6 (11.8)	4 (9.3)	0.74

Hypertension	5 (50.0)	18 (20.7)	11 (21.6)	10 (23.3)	0.22
Proteinuria	4 (40.0)	19 (21.8)	10 (19.6)	5 (11.6)	0.20
Thrombosis	0	2 (2.3)	2 (3.9)	6 (14.0)	0.03

Notes: [a]graded as per the National Cancer Institute Common Terminology Criteria for Adverse Events (CTCAE)

Abbreviations: trAE – treatment-related adverse event.

Supplementary Figure 1. BMI association with baseline patient characteristics. *p* values shown for Pearson correlation coefficient for continuous variables and unpaired student t-test for categorical variables. Mean BMI (square) with 95% confidence interval (green) shown for categorical variables.

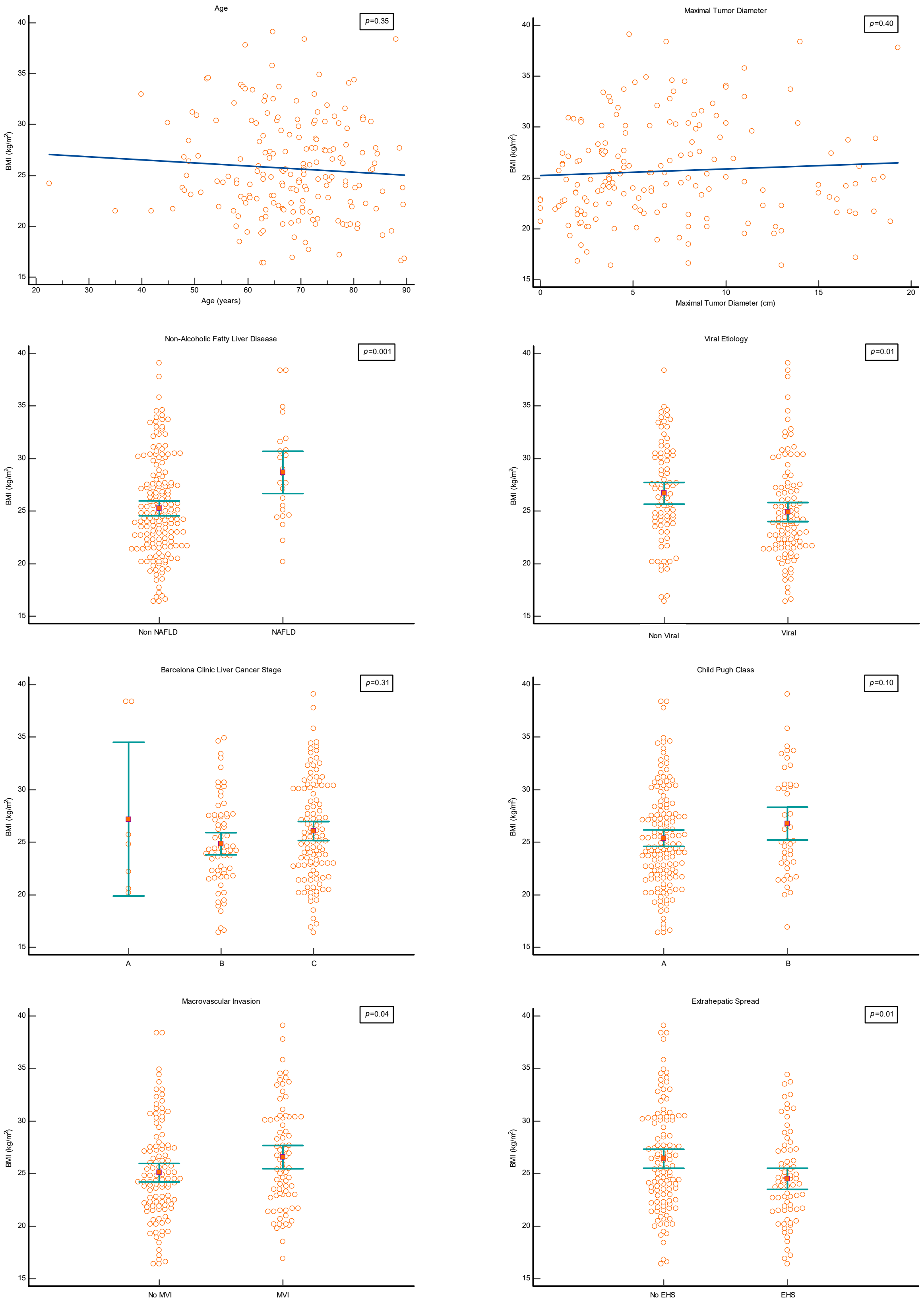

Supplementary Figure 2. Kaplan-Meier curve showing overall survival (months) for underweight (BMI < 18.5), normal (18.5 ≤ BMI < 25), overweight (25 ≤ BMI < 30) and obese (BMI ≥ 30) patients with unresectable hepatocellular carcinoma patients after atezolizumab plus bevacizumab administration.

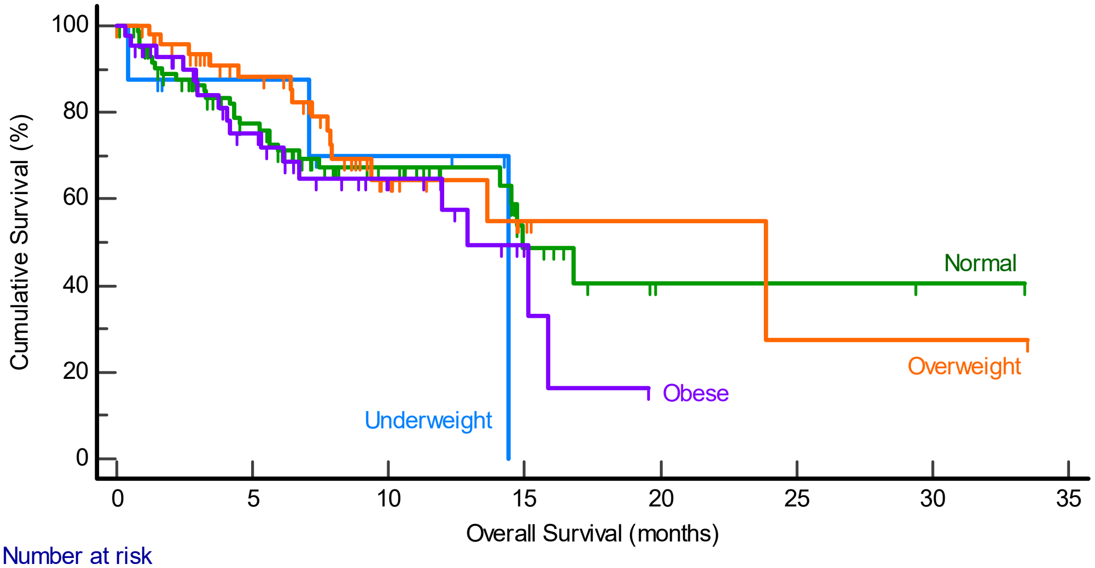

Number at risk

	0	5	10	15	20	25	30	35
Underweight	8	5	3	0	0	0	0	0
Normal	85	50	25	9	2	2	1	0
Overweight	50	32	11	4	2	1	1	0
Obese	43	24	11	3	0	0	0	0

Supplementary Figure 3. Kaplan-Meier curve showing progression-free survival (months) for underweight (BMI < 18.5), normal (18.5 ≤ BMI < 25), overweight (25 ≤ BMI < 30) and obese (BMI ≥ 30) patients with unresectable hepatocellular carcinoma patients after atezolizumab plus bevacizumab administration.

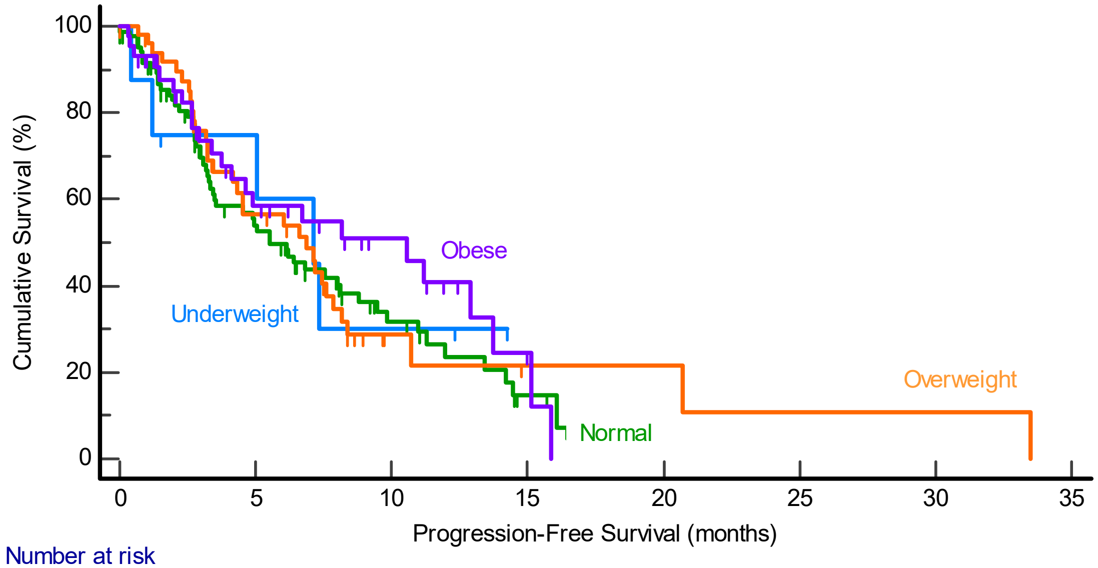

Number at risk

Group	0	5	10	15	20	25	30	35
Group: Underweight	8	5	2	0	0	0	0	0
Group: Normal	84	38	14	3	0	0	0	0
Group: Overweight	50	23	4	2	2	1	1	0
Group: Obese	43	19	10	2	0	0	0	0
